# Redefining Body-Self Relationships Through Outdoor Physical Activity: Experiences of Women Navigating Illness, Injury, and Disability

**DOI:** 10.3390/bs15081006

**Published:** 2025-07-24

**Authors:** Joelle Breault-Hood, Tonia Gray, Jacqueline Ullman, Son Truong

**Affiliations:** 1 School of Education and the Centre for Educational Research, Western Sydney University, Penrith, NSW 2751, Australia; 2 World Leisure Centre for Excellence (WLCE), Western Sydney University, Penrith, NSW 1797, Australia; 3 School of Health and Human Performance, Dalhousie University, Halifax, NS B3H 4R2, Canada

**Keywords:** women, positive body image, outdoor physical activity, outdoor recreation, illness, injury, disability, mental health, health, wellbeing

## Abstract

Physical challenges such as illness, injury, and disability significantly alter women’s relationships with their bodies, disrupting established notions of functionality and self-worth. This study re-examines the Holistic Model of Positive Body Image and Outdoor Physical Activity through secondary analysis focusing on women with illness, injury, and disability. From the original sample of *N* = 553 female participants, open-ended survey responses were identified from *n* = 84 participants (15.2%) who self-disclosed as having illness, injury, or disability to examine how outdoor settings facilitate positive body image. Through reflexive thematic analysis, the study revealed three key mechanisms: (1) personalized redefinition of functionality transcending standardized metrics, (2) therapeutic engagement with natural environments fostering embodied acceptance, and (3) cyclical reinforcement between physical capability and psychological wellbeing. The findings confirm the model’s utility while indicating necessary adaptations to address the fluctuating nature of body functionality. The adapted model emphasizes how outdoor recreational activities create contexts for reimagining body-self relationships across the spectrum of physical experiences—from temporary recovery to ongoing adaptation of persistent conditions—with implications for rehabilitation professionals, outdoor educators, and healthcare providers.

## 1. Introduction

When our bodies change through illness, injury, or disability, so does our relationship with ourselves. The associated physical transitions may significantly alter women’s orientations toward their bodies, challenging established notions of functionality, capability, and self-worth (Crooks, 2016; Rice et al., 2015; Thomas et al., 2019). Globally, approximately 16% of society is living with some form of disability (World Health Organization, 2023). Previous research has explored connections between outdoor activity and positive body image (Breault-Hood et al., 2017; Mitten & D’Amore, 2017), but we face a critical gap: how do women experience these outdoor spaces during or after physical challenges? Outdoor environments may offer unique qualities—sensory richness, absence of performance metrics, freedom from judgmental gazes—that create distinctive possibilities for women to reclaim agency during physical transitions.

Research has established promising relationships between time spent in natural environments and positive body image (Bacevičienė et al., 2021; Jankauskiene et al., 2022; Swami et al., 2016, 2018, 2019, 2020), with physical activity in nature mediating this relationship (Bacevičienė et al., 2021). Despite these insights, there remains no established theory explaining the relationship between body image and physical activity (Martin-Ginis et al., 2012), which is a gap more pronounced when considering diverse embodiments. In this paper, we use the term “diverse embodiments” to encompass the full spectrum of lived bodily experiences among women with illness, injury, and disability, acknowledging the varied ways these individuals inhabit, experience, and engage their bodies in the world. Embodiment refers to the “lived experience of engagement of the body in the world” (Piran & Teall, 2012, p. 171), offering a lens through which we can explore and honor these varied bodily realities and their significance.

**Figure 1:**
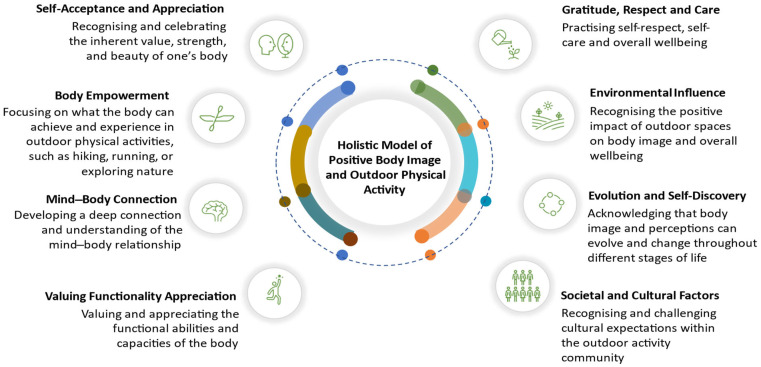
The Holistic Model of Positive Body Image. (Sourced from Breault-Hood, 2023).

This paper extends Breault-Hood’s (2023) Holistic Model of Positive Body Image and Outdoor Physical Activity (Figure 1) by examining how its eight principles apply to women experiencing physical challenges. Through secondary analysis of data from participants who identified as having an illness, injury, or disability in the original study (*N* = 84), we interrogate each principle of the model through the lens of diverse embodiments. This approach centers the voices of women navigating physical challenges, enriching theoretical understanding and informing more inclusive approaches to outdoor engagement.

## 2. Literature Review

### 2.1. The Transformative Potential of Nature Immersion and Outdoor Physical Activity

Research increasingly recognizes how physical activities in natural environments reshape bodily relationships. Piran’s (2017) work shows physical activity fosters agency and embodied connection, while Tylka and Piran (2019) established positive body image as multidimensional, extending beyond mere absence of dissatisfaction to include active appreciation and respect for the body. Outdoor settings offer powerful contexts for positive body image development. Mitten and D’Amore (2017) found that immersion in natural environments during physical activity created unique opportunities for women to experience their bodies as capable.

Participation in outdoor activities amongst like-minded women fosters resilience during challenging periods (Buckley & Westaway, 2020; Chen et al., 2024; Houge Mackenzie et al., 2021; Pomfret et al., 2025). Recent findings confirm outdoor recreation enhances mental well-being, boosting coping mechanisms and reducing stress (Pomfret et al., 2024). Women who regularly trail run experience strong community, increased self-confidence, enhanced adaptability, and improved mental well-being (Lincoln, 2021). Women increasingly pursue restorative experiences in nature to counteract frenetic lifestyles and seek meaning (Alizadeh & Filep, 2023; Pomfret et al., 2024).

Research provides insight into how outdoor physical activity supports positive body image. Research provides insight into how outdoor physical activity supports positive body image. Alleva et al. (2020) and Alleva and Tylka (2021) found functionality-focused interventions enhanced body appreciation while reducing appearance concerns. Tylka and Homan (2015) revealed exercise motivated by functional goals rather than appearance is associated with greater intuitive eating and body appreciation. Yet, most outdoor activities remains designed for normative bodies (Seaman et al., 2014). The specific mechanisms of nature exposure on body image require examination, as different natural environments may have varying effects, with blue spaces potentially offering unique benefits compared to green spaces (Rygal & Swami, 2021; Stieger et al., 2022). Sundgot-Borgen et al. (2022) found self-compassion and nature connectedness together explained substantial variance in body appreciation. These relationships remain underexplored for women navigating illness, injury, or disability.

### 2.2. Bodies, Narratives, and Identity During Physical Transitions

Sociocultural theories show how beauty standards become internalized and shape bodily self-evaluations (Fredrickson & Roberts, 1997; Tiggemann, 2011). For women with illness, injury, or disability, these standards intersect with ableist expectations, further marginalizing diverse embodiments (Moss & Dyck, 2003; Rice et al., 2021). Garland-Thomson’s (2011) “fitting” and “misfitting” framework reveals how embodied experiences form through relationships between bodies and environments, creating connections either harmonious or disconnected based on “the degree to which that shared material world sustains the particularities of our embodied life” (Garland-Thomson, 2011, p. 596). Thomas et al. (2019) emphasize that body functionality research “should recognize what some bodies cannot do, and that many bodies function differently” (p. 89).

For women in outdoor activities, illness, injury, or disability that changes physical capabilities demands significant identity work. Allen-Collinson (2017) notes that “the assault on identity generated by illness, pain, and injury has been well documented” (p. 267), though it remains underexplored in non-elite contexts. When bodies change—temporarily, permanently, cyclically—women must negotiate “disrupted body projects” (p. 268). This disruption is not merely physical, but existential. For women whose sense of self forms partly through outdoor engagement, physical challenges threaten both functional capabilities and core aspects of identity and belonging. The task becomes building new narratives about bodies, capabilities, and worthwhile navigating sociocultural pressures that render non-normative bodies invisible or problematic.

Research examining body image during illness and injury recovery reveals distinct psychological processes. Charmaz’s (2002) work shows how unpredictable symptoms create a ‘loss of self’ by disrupting established body image. Papathomas et al. (2015) found challenges in temporary limitations—participants struggled to reconcile former athletic identities with present limitations while projecting toward recovery. This narrative reconstruction differs following injury, where individuals experiencing temporary challenges seek a return to familiar stories, while those facing permanent conditions craft new narratives of identity and capability (Sparkes & Smith, 2013).

Feminist materialist disability theory presents promising avenues for expanding body functionality literature (Rice et al., 2021). By reconsidering “what a body can do,” this approach enriches conceptualizations of functionality in positive body image research. This perspective avoids prescribing what gendered, raced, or disabled bodies inherently are or do, instead acknowledging how social conventions, practices, and discourses shape physical capacities and materiality. It envisions the body as fundamentally dynamic, not fixed, but continually “becoming” (Rice et al., 2021). This framework operates bidirectionally by looking forward to analyzing conditions that might broaden bodily potential, while examining evolving meanings of concepts that inform contemporary understandings.

### 2.3. Reimagining Bodies in Outdoor Contexts: Beyond Deficit Models

Alternative frameworks offer more empowering approaches to diverse embodiments. Garland-Thomson’s (2011) concept of misfitting shifts attention from individual broken bodies to environments designed without diverse physical bodies in mind. For women experiencing illness or injury, these misfits may be temporary or fluctuating, creating what Price (2015) describes as complex bodymind experiences—integrated physical and cognitive realities that challenge conventional frameworks and resist categorization. “Bodymind” resists the mind/body split in Western thought that privileges the mind and treats mind and body as separate entities (Clare, 2017; Hendren, 2020). It reconfigures human embodiment as fluid and indivisibly entangled with the world, situating embodied experience as knowledge. Bodymind differences refer to embodied non-normatives or bodies and minds that westernized cultures define as outside “normal” (Clare, 2017).

Positive body image frameworks offer promising avenues for understanding this reimagining. Piran’s (2017) developmental theory of embodiment emphasizes connection, agency, and freedom from objectification. For women experiencing physical challenges, these frameworks raise critical questions: What happens to functionality appreciation when what a body can do changes day by day? How might body acceptance be practiced when bodies feel unpredictable?

### 2.4. Research Gaps and Current Study

A critical limitation persists in the current research landscape: empirical studies have predominantly focused on women with normative bodies, creating significant conceptual and methodological gaps. Traditional quantitative measures developed with normative bodies in mind often fail to capture complex, fluctuating experiences (Bailey et al., 2016). Researchers have begun exploring body functionality among diverse populations. Thomas et al. (2019) examined how women with visible physical disabilities experience body functionality, while Tabaac et al. (2018) addressed intersectional perspectives on body image among transgender and gender-nonbinary individuals. However, these studies have not specifically examined how outdoor environments might mediate these experiences. To address this gap, the present study investigates how women with illness, injury, and disability experience outdoor physical activity, and how these experiences shape their body-self relationships.

## 3. Methodology

### 3.1. Research Design

This work takes up a secondary analysis (Heaton, 2008; Sherif, 2018) that returns to a dataset originally collected by Breault-Hood (2023) examining the Holistic Model of Positive Body Image and Outdoor Physical Activity. Secondary analysis creates possibilities for questions not imagined in original study designs, extending the impact of rich qualitative data. The original data collection was conducted through Qualtrics surveys distributed via Facebook groups and snowball sampling. From 553 female-identifying participants came multiple forms of data: structured responses, scaled measurements, and open-ended survey responses.

This secondary phase of analysis identified women whose experiences included illness, injury, disability, or recovery processes through a thorough review of open-ended responses. This screening process identified 84 women (15.2% of the original sample) whose responses described significant experiences with physical challenges. Their physical transitions varied widely: from acute injuries and surgical recoveries to chronic conditions, including autoimmune disorders, multiple sclerosis, and fibromyalgia; cancer diagnosis and treatment; pregnancy and birth-related physical changes; age-related transitions; temporary and permanent disabilities; and fluctuating conditions.

### 3.2. Participant Characteristics

Participants were predominantly middle-aged, with over half (52.4%) in the 45–64 age range. A striking difference emerged in employment status, with nearly a third (32.1%) reporting being unable to work—compared to only 3.8% in the overall sample. Despite this employment disparity, 82.2% described their finances as “moderately well” or “very well.” What proved especially significant is how these women engaged with outdoor spaces despite their physical challenges: 60.8% participating 2–4 times per week or more frequently (compared to 43.7% in the overall sample), predominantly at moderate intensity (75.0%). Notably, 82.1% reported that their body perception had changed positively through outdoor physical activity, suggesting that natural environments offered meaningful contexts for reimagining body-self relationships despite physical challenges.

#### 3.2.1. Sampling Limitations and Transferability Considerations

The 84 women included in this analysis represent those who self-disclosed as living with illness, injury, or disability in their open-ended survey responses, rather than a purposively recruited sample. The sample is limited by the characteristics of the original dataset, including recruitment through social media channels and an age distribution skewed toward middle-aged women (52.4% aged 45–64). Additionally, the women included in this analysis reported relatively high levels of ongoing outdoor activity engagement (60.8% participating 2–4 times per week or more), suggesting they may represent a subset of women with physical challenges who have maintained positive relationships with outdoor environments. This potentially excludes experiences of women who have discontinued outdoor engagement due to their conditions or who face greater barriers to participation.

Transferability may therefore be limited to younger women, those from different cultural contexts, those recruited through clinical settings, or those who have had more negative experiences with outdoor activity during physical challenges. The findings should be understood as representing one segment of experiences rather than the full spectrum of how women with illness, injury, or disability relate to outdoor environments.

#### 3.2.2. Condition Categories and Temporal Dimensions

The analysis revealed distinct patterns based on condition type and temporal characteristics, which we categorized as follows: 

(1) Acute/temporary conditions (*n* = 23, 27.4%): Post-surgical recovery, injury rehabilitation, and acute illness episodes. These participants often focused responses on “returning to normal” and expressed frustration with temporary limitations while maintaining hope for full recovery.

(2) Chronic/fluctuating conditions (*n* = 41, 48.8%): Fibromyalgia, multiple sclerosis, long COVID-19, autoimmune disorders, and chronic pain conditions. These women developed sophisticated adaptation strategies, describing cycles of capability and limitation that required ongoing negotiation with their bodies and outdoor environments.

(3) Permanent/stable conditions *(n* = 20, 23.8%): Amputation, spinal cord injury, permanent disability from injury. These participants engaged in fundamental identity reconstruction, often developing entirely new frameworks for understanding functionality and relationships with outdoor spaces.

Analysis revealed distinct patterns: women with temporary conditions often focused on restoring their previous state,’ while those with chronic conditions developed sophisticated adaptation strategies, and those with permanent conditions engaged in fundamental identity reconstruction. This temporal dimension significantly influenced how participants experienced and described their relationships with outdoor environments.

### 3.3. Analytical Framework

#### 3.3.1. Development of the Original Model

The Holistic Model of Positive Body Image and Outdoor Physical Activity emerged in response to limitations in existing body image frameworks, which have historically overlooked outdoor physical activity contexts and women’s experiences. The model builds upon Wood-Barcalow et al.’s (2010) conceptualization of positive body image, extending the model by explicitly centering the outdoor environment as a mediating factor in women’s body image development. Theoretical foundations for this approach draw from Piran’s (2017) Developmental Theory of Embodiment, particularly its dimensions of body connection/comfort and agency/functionality. Empirical support for the model’s focus comes from recent research, with Mitten and D’Amore (2017) finding that immersion in natural environments creates unique opportunities for women to experience their bodies as capable rather than decorative.

Methodologically, the model was developed through a convergent mixed-methods approach, integrating quantitative findings and qualitative insights from thematic analysis. Specifically, quantitative data established significant correlations between outdoor activity frequency and both body appreciation and functionality appreciation. The model articulates eight interconnected principles (shown in Figure 1) that collectively address self-acceptance, body empowerment, mind-body connection, functionality appreciation, environmental influence, and sociocultural contexts as they relate to positive body image development through outdoor physical activity.

#### 3.3.2. Theoretical Framework for Disability-Informed Reinterpretation

This secondary analysis positions itself at the intersection of disability studies and women’s outdoor experiences—two areas rarely examined together. While the original model centered women’s experiences, it did not explicitly address how disability might mediate these experiences. This gap reflects broader trends where diverse embodiments are often rendered invisible in outdoor programming (Laurendeau et al., 2020). This study is positioned as a model extension that examines the boundaries of the original framework. Following Kafer’s (2013) argument that disability perspectives can ‘crip’ or ‘reimagine’ existing theoretical models, this analysis asks how the experiences of women with illness, injury, or disability might enrich or challenge The Holistic Model of Positive Body Image and Outdoor Physical principles.

### 3.4. Data Analysis Procedures

Reflexive thematic analysis (Braun & Clarke, 2019) guided the data analysis process, providing both methodological structure and interpretive flexibility. Following this analytical approach, Breault-Hood’s (2023) eight principles were used as sensitizing concepts while remaining open to unexpected patterns across 84 open-ended survey responses about embodied experience.

The analytical process followed Braun and Clarke’s (2019) thematic analysis methodology through six phases:

Familiarization with data: The lead author began by reading all 84 open-ended survey responses multiple times to gain deep familiarity with the data, making initial notes about recurring patterns and potential themes specific to physical challenges.

Generating initial codes: Initial coding began with line-by-line analysis of each response, using both deductive codes derived from the eight principles and inductive codes emerging from participant experiences. A detailed coding framework was developed, including codes such as “redefined capability,” “cyclical adaptation,” “embodied expertise,” and “therapeutic landscape engagement.”

Developing potential themes: Initial codes were grouped into potential themes, maintaining awareness of both alignment with and departure from the original model.

Reviewing themes against data: Themes were reviewed by returning to the original survey responses, checking for coherence within themes, and clear distinctions between themes. During this phase, we specifically sought out “negative cases” that might challenge emerging interpretations.

Defining final themes: Themes were refined and clearly defined, with particular attention to how principles from the original model were transformed by experiences of illness, injury, and disability.

Producing analysis: The final analysis was written with attention to the dialectical relationship between the original model and how women with physical challenges experienced and described their body-self relationships in outdoor contexts through their survey responses.

The eight principles from the original model served as sensitizing concepts (Bowen, 2006)—not rigid categories but attentional guides. To enhance rigor, the ongoing analysis was discussed collaboratively amongst the research team to refine the development of the codes and themes throughout the process. Analytical decisions were documented in a reflexive journal throughout the process.

For this reinterpretation, we draw on feminist disability theory (Garland-Thomson, 2011; Hall, 2011) and critical embodiment perspectives (Rice et al., 2021; Thomas et al., 2019). This theoretical framing acknowledges that the original model primarily reflected experiences of normative bodies. As Wendell (2001) notes, traditional body image frameworks often presume stable, predictable embodiment—an assumption challenged by the fluctuating nature of many illnesses and disabilities.

### 3.5. Researcher Positionality and Reflexivity

Researchers engaging with experiences of physical challenge are influenced by their own embodied experiences, which shape how they approach, interpret, and represent these accounts. The research team brings the lived experience of multiple physical challenges to this analysis. For instance, the lead researcher has endured spinal surgeries and recovery from brain tumor resection, resulting in facial paralysis, vestibular issues, and single-sided deafness. These experiences have provided an understanding of both temporary and permanent physical adaptations. This insider positioning offers both strengths and potential limitations. The researchers’ lived experiences allow for recognition of nuances in how women describe navigating physical limitations, while requiring vigilance against universalizing individual experiences. Throughout the analysis, a reflexive stance was maintained, documenting how bodily experiences informed interpretations while noting where participants’ experiences diverged from the researchers’ own. The analysis was approached with an awareness of the danger of imposing a single narrative of physical challenge onto diverse experiences. Following feminist disability scholarship, the research centered on the embodied knowledge of women navigating physical challenges while remaining alert to how researchers’ own experiences might both enhance understanding and potentially limit it.

### 3.6. Ethical Considerations

The primary data collection received approval from the Human Research Ethics Committee at Western Sydney University (HREC Approval Number: H11143). This secondary analysis was conducted under the original HREC approval, with additional ethical protocols for focusing on women with disabilities. The lead author maintained analytical independence by: (1) developing new research questions not addressed in the original study, (2) employing different theoretical frameworks (feminist disability theory), and (3) following systematic analytical procedures documented through reflexive journaling. All participants provided informed consent for potential secondary analysis of their data. All data remained anonymized, with pseudonyms used throughout. Given the potentially sensitive nature of accounts about physical challenges and body image, we approached the analysis with enhanced sensitivity to respect participants’ experiences while protecting their privacy.

## 4. Findings: Bodies That Pause, Bodies That Persist

For this secondary analysis, we isolated responses from the original sample of *N* = 553 female participants, focusing specifically on the 84 participants (15.2%) who identified as having an illness, injury, or disability. Using reflexive thematic analysis guided by Braun and Clarke’s (2019) six-step approach, we re-examined these participants’ qualitative responses through the framework of Breault-Hood’s (2023) Holistic Model of Positive Body Image and Outdoor Physical Activity.

Our analysis reveals not merely differences but divergent orientations: bodies temporarily displaced from familiar capabilities versus bodies permanently reoriented to alternative possibilities. As Ahmed (2006) notes, illness, injury, and disability fundamentally alter how we “reside in space” (p. 1), creating “moments of disorientation” (p. 157) that require new ways of inhabiting both bodies and environments. Through this disability-informed lens, the eight principles from the original model transform in significant ways, coalescing around three key mechanisms that illuminate how participants with physical challenges experience their body-self relationships in outdoor contexts.

### 4.1. Personalized Redefinition of Functionality Transcending Standardized Metrics

When bodies change through illness or injury, traditional measures of functionality become inadequate. Participants in this study developed sophisticated understandings of what their bodies could do that moved far beyond standardized metrics of distance, speed, or technical difficulty. This redefinition represents both resistance to normative expectations and creative adaptation to changing capabilities.

#### 4.1.1. Agency Through Embodied Expertise

Body empowerment shifts from dominance or control to what Allen-Collinson (2017) calls disrupted body projects—the sustained effort to maintain continuity when physical capabilities change. Among our participants, agency manifested not as command but as conversation, as negotiating with rather than controlling the body. Participants developed what Crooks (2016) terms embodied expertise, choreographing movements within new constraints.

Participants recovering from acute illness or injury described reclaiming agency through linear trajectories—a gradual return to familiar capability. However, for those with chronic conditions, our data revealed that agency manifested differently: as adaptation, as and pacing, as finding alternate routes to desired experiences.

A participant who experienced cancer reflected: “I had cancer 11 years ago and spent a year on crutches. I will never ever take my health or my mobility for granted again. Every day I am able to move is a gift and opportunity.” Here, motion itself transforms from expectation to gift. This aligns with what Pini and Soldatic (2012) describe as the “material and discursive reconstitution” (p. 386) of the embodied self—where the taken-for-granted becomes the newly appreciated.

For participants with chronic conditions, agency took on more complex dimensions. A participant with fibromyalgia explained:

“I have fibromyalgia so must exercise frequently and at moderate pace to keep healthy and avoid muscle flare ups and extreme fatigue. I’m incredibly grateful for what my body can do because I’ve seen the other side of the spectrum.”

Note the specialized knowledge embedded in this account—what Crooks (2016) terms “embodied expertise.” This participant has learned to navigate not despite, but through her condition, developing what might be called a choreography of care—movements carefully calibrated to maintain rather than exhaust capacity.

#### 4.1.2. Redefining What Function Means

Valuing functionality appreciation expands beyond celebrating what bodies can do to embrace what Thomas et al. (2019) identify as the complex, fluctuating nature of functionality for diverse embodiments. Function becomes not an absolute measure but a creative negotiation—acknowledging both capability and limitation, adaptation and persistence.

A central theme in our participants’ responses was the process of redefining what functionality means after experiencing physical limitations. Participants with temporary injuries often focused on returning to previous functionality, while those with permanent disabilities developed entirely new frameworks for understanding functionality. One participant with a prolapse after childbirth exemplified this redefinition:

“Post babies, I have a new perspective on my body. Having had a birth injury resulting in pelvic organ prolapse, I am mindful that my body does not function the same as it used to, but I’m also amazed at how I have adapted and can still do my favourite outdoor activity (mountain biking).”

Notice the coexistence of acknowledgment “does not function the same” and amazement “how I have adapted”. This response illustrates Thomas et al.’s (2019) argument that for women with physical challenges, body functionality encompasses not only what bodies can do, but also what they cannot do or do differently. The participant celebrates adaptation rather than focusing solely on limitation.

Another participant described her changed relationship with functionality after hip surgery:

“I’ve had reduced functionality following one total hip replacement and ahead of another. This has been frustrating and I’m still in a level of regular pain. Nonetheless I’m back outdoors regularly because being outdoors makes me happy, and I have less years ahead than I’ve had before so I need to get moving!!”

The tension between “reduced functionality” and being “back outdoors” opens a space for questioning: what constitutes “function” in outdoor contexts? Is it pace, distance, or difficulty of terrain? Or might it be presence itself—the being in natural spaces regardless of how that being occurs? Our participants’ responses demonstrate the creative adaptability that women develop when navigating changed bodies, moving beyond binary understandings of ability/disability to embrace what remains possible.

### 4.2. Therapeutic Engagement with Natural Environments Fostering Embodied Acceptance

Natural environments emerged as uniquely supportive spaces for women navigating physical challenges, offering qualities that built environments could not provide. This therapeutic engagement operated through multiple dimensions: sensory restoration, freedom from judgment, and opportunities for embodied acceptance.

#### 4.2.1. Self-Acceptance as Ongoing Practice

When bodies change through illness or injury, self-acceptance and body appreciation become not a peaceful reconciliation but an ongoing labor—what Charmaz (2002) describes as navigating the loss of self that accompanies physical transitions. For participants in this study, self-acceptance operated as a continual practice of befriending a body that may feel unfamiliar, of making a home in altered terrain.

Participants with acute injuries identified patience as central to the process of self-acceptance—a temporary suspension of expectation, a waiting for return. But for those with chronic conditions or disabilities, our data showed that acceptance became something more profound: not waiting for but dwelling with, not recovery but discovery of alternative ways of being in bodies. A participant diagnosed with endometriosis wrote:

“The last two years I have struggled more than usual—I was diagnosed with endometriosis which has led to weight gain but also to not being able to use my body in the ways I have done for most of my life. I get tired, and sore, and I’m scared of making things worse or over-exerting myself. I am sometimes angry at my body for not being the reliable friend it has always been.”

Notice the relational language—the body as “friend,” albeit an unreliable one. This reveals what Thomas et al. (2019) term disrupted embodiment, where the body becomes simultaneously self and other. Self-acceptance here is not the absence of negative emotion, but its incorporation into a more complex relationship with embodiment.

For a participant with multiple sclerosis, this labor produces a different orientation: “I have a neurological disease, so even though I have body image issues, I am extremely grateful that I have a fully functioning body.” The conjunction “even though” marks a space of tension between “image issues” and “gratitude,” and between social ideals and material function.

#### 4.2.2. Enhanced Mind-Body Awareness

The mind-body connection deepens through necessity—what was once optional becomes essential. Our analysis found that physical limitations created heightened body awareness among participants, a specialized attunement to signals and sensations. The body becomes not an object to control but a source of knowledge to heed, particularly in unpredictable outdoor environments. A participant recovering from injury reflected:

“I am very aware of what my body needs to function well, and I am more inclined to rest now rather than push through and cause injuries if I am not feeling the best. It has taken a long time for me to be content with the body I have been given. I am much kinder and engage in more low-impact activities, lots of massage, and recover now too.”

The phrase “I am much kinder” invites us to consider: what does kindness toward one’s body mean in a culture that often celebrates pushing through pain? This enhanced mind-body connection reflects what Tylka and Piran (2019) describe as body attunement—an essential component of positive body image that involves listening and responding to bodily needs and sensations.

#### 4.2.3. Nature as Therapeutic Landscape

Environmental influence gains new resonance through Garland-Thomson’s (2011) concept of misfitting—the productive friction between diverse embodiments and spaces designed for normative capabilities. Natural environments offer what built spaces often cannot: freedom from standardized metrics, from judgmental gazes, and from prescribed ways of moving.

The meeting between diverse embodiments and natural environments creates distinctive possibilities for healing and adaptation. Participants frequently described outdoor settings as uniquely supportive during physical challenges—as if nature offered different possibilities for bodies in transition than built environments dominated by normative expectations. A participant who survived cancer described her experience:

“I underwent chemo and radiation ten years ago… I would go into radiation at 9:00 and then go to a trail he suggested… Although my body was sick and buttered by the intense treatment, being outside helped me regain my sense of self.”

The phrase “regain my sense of self” suggests outdoor environments offered not just physical but ontological restoration—spaces where identity disrupted by illness might be reclaimed. For participants managing chronic conditions, natural settings offered distinct respite: “I know whenever I am super low I can go to nature to bring myself back up. At least once a year I take a solo camping/hiking trip to reconnect myself to nature.” Nature becomes not just a location, but an agent in recovery—a presence that actively participates in restoration.

### 4.3. Cyclical Reinforcement Between Physical Capability and Psychological Wellbeing

Unlike linear models of recovery or adaptation, participants described cyclical processes where physical engagement with outdoor environments reinforced psychological well-being, which in turn supported continued physical participation. This cyclical relationship was particularly evident among women with chronic conditions who experienced fluctuating capabilities.

#### 4.3.1. Gratitude Emerging Through Limitation

Gratitude and respect take on heightened significance through experiences of limitation. Our analysis revealed that among participants, gratitude emerges not despite constraint, but through it—a recognition of the body’s persistent gifts even when changed. Motion transforms from expectation to gift; capability from right to privilege.

Many participants expressed how experiences of illness, injury, or disability fostered deeper gratitude for their bodies—participants with temporary injuries often expressed gratitude for recovery processes, while those with permanent conditions developed appreciation for specific capabilities that remained intact. A participant with MS reflected:

“Having MS, osteoporosis and T12 compression fracture, I’m grateful for anything my body allows me.” The phrase “anything my body allows me” reveals a shifted orientation—from body as possession to body as granting permission, from capabilities as rights to capabilities as gifts.

Another participant who had experienced cancer shared:

“My body has experienced a life-threatening illness, and as a result, it is quite different than it used to be. And yet, I can do all that I do, and my body enables me to do it. How could I not love it?”

The rhetorical question suggests transformation not just in body but in relationship to body. This participant has moved from an aesthetic to a functional valuation, from body as visual object to body as enabling presence.

#### 4.3.2. Transformation Through Challenge

Evolution and self-discovery are reframed through participants’ experiences of physical challenge as catalysts for growth. Rather than viewing illness or injury solely as disruptions to normal functioning, many participants integrated these experiences into expanded understandings of their bodies’ capabilities and worth. One participant reflected on how her outdoor experiences changed during long COVID-19:

“My relationship has changed a lot with my body in the last 6 months. It’s really frustrating not to do the activities I want to, but I know my body is working hard to fight the fatigue and inflammation from covid. It’s still my friend, even if I’m disappointed in it!”

This personification of the body as a “friend” despite disappointment aligns with what Allen-Collinson (2017) describes as the identity work required to maintain continuity in self-concept when physical capabilities change. The participant reconstructs her relationship with her body as a partnership rather than ownership.

Another participant described the transformation that occurred after experiencing temporary vision loss:

“For a period, I only had 20% vision and couldn’t drive (later this was resolved with surgery). It was an interesting experience. I found numerous ways to compensate and manage with poor vision. I found I didn’t miss my sight much, and I didn’t fear loss of sight, instead I found new ways of living opening up using all my other senses, and I learned to respect those with real disabilities much more.”

This response illustrates how physical challenges can lead to expanded perspectives, new skills, and increased empathy—transformations that extend beyond the physical body itself.

#### 4.3.3. Community and Social Navigation

The social dimensions of navigating illness, injury, and disability in outdoor settings emerged as crucial to maintaining this cyclical reinforcement. Participants with temporary injuries often received more readily accessible support, while those with chronic conditions faced greater challenges in finding appropriate communities and navigating inconsistent capabilities. One participant with a disability highlighted:

“I have a mixed relationship with my body’s capabilities. Sometimes I am amazed at what it can do and appreciate its strength and resilience. I do however have an autoimmune condition, so sometimes my body needs rest or isn’t capable of doing things some would perceive as everyday tasks. It is very up and down but overall I appreciate that I can still get out and do hikes etc. when I am well.”

This response highlights the fluctuating nature of many conditions and the importance of community understanding of these variations. As Crooks (2016) emphasizes, women with chronic conditions must navigate not only their changing capabilities but also social expectations that presume consistent functionality. Another participant reflected on social barriers and expectations:

“I am overweight and have various health issues but feel proud and empowered undertaking the vigorous activities I do. I love that people judge the way I look and are then surprised the level of fitness I have and what I can achieve.”

For many participants in our study, finding communities that focused on capability rather than appearance was crucial for maintaining outdoor engagement during and after recovery processes.

## 5. Discussion

### 5.1. Cyclical Rather than Linear: A Reconceptualized Model

The thematic analysis revealed that the eight principles of Breault-Hood’s (2023) original model operate through distinct mechanisms when applied to women with physical challenges. Rather than following a linear progression, these women often described cyclical processes of adaptation, setback, and renewed engagement. This cyclical nature is particularly pronounced for those with chronic conditions, who must continuously renegotiate their relationship with outdoor environments as their capabilities fluctuate. This adapted model extends beyond the original framework by explicitly addressing the temporal dimensions of physical challenge—recognizing that women’s relationships with their bodies evolve differently based on whether they are navigating temporary injuries, progressive conditions, or stable disabilities.

The findings suggest that outdoor environments offer distinctive contexts for this negotiation—spaces where bodies in transition might discover new possibilities for meaning, movement, and belonging. Rather than positioning adaptation as a compromise, the experiences of these 84 women reveal it as a form of embodied wisdom that transforms limitation into a site of potential.

### 5.2. Bodies Finding New Ways to Dwell

The findings extend existing understanding of the relationship between outdoor physical activity and positive body image by specifically examining the experiences of women navigating illness, injury, and disability. Several key insights emerge from this analysis: First, outdoor physical activity provides distinctive opportunities for women to reconcile changed or changing physical capabilities with their sense of identity. Unlike many indoor exercise contexts that emphasize standardized performance metrics or aesthetic outcomes, outdoor environments allow for more personalized definitions of achievement and success. The mountain that once was climbed quickly might still be climbed slowly; the trail once run might now be walked; the view once taken for granted might become newly significant when reached through different means.

Second, the natural environment itself plays a therapeutic role in recovery processes. Participants’ narratives suggest that outdoor settings offer sensory experiences, perspective shifts, and freedom from judgment that are particularly valuable during periods of physical vulnerability. Natural spaces—with their absence of mirrors, performance measurements, and standardized equipment—create room for bodies to move differently, to pause when needed, and to find their own rhythms of engagement. This finding extends existing research on nature’s psychological benefits (Bratman et al., 2021), highlighting its specific relevance to body image during recovery.

Third, the study reveals the iterative relationship between physical recovery and psychological well-being. Participants’ accounts demonstrated how outdoor engagement facilitated both physical rehabilitation and psychological adjustment to changed bodies or capabilities. The physical challenge of navigating uneven terrain becomes simultaneously a psychological process of redefining capability; the sensory experience of wind on skin becomes a reminder of embodied presence despite limitation. This suggests that outdoor programs may offer unique benefits for holistic recovery approaches that address both physical and psychological dimensions of healing.

Finally, the findings highlight women’s resilience in redefining their relationships with their bodies through physical challenges. Rather than viewing illness or injury solely as disruptions to normal functioning, many participants integrated these experiences into expanded understandings of their bodies’ capabilities and worth. This reframing represents a form of resistance against limiting cultural narratives about disability, aging, and women’s bodies. The women in this study did not merely adapt to limitation but transformed their understanding of what bodies are for—moving from decoration to function, from standardized performance to personalized experience, and from body as possession to body as companion.

### 5.3. Practical Implications

This study offers several important practical implications for professionals working with women experiencing physical challenges in outdoor contexts. For outdoor program providers, findings suggest a need to move beyond normative physical capability assumptions that exclude those with illness, injury, or disability (Wilson, 2017). Women’s experiences of “functionality” during physical challenges are highly personalized and fluctuating, calling for what Thomas et al. (2019, p. 91) term a “capability-sensitive approach”—offering multiple route options, flexible pacing, and redefining “success” beyond distance or technical difficulty.

Drawing on Crooks’ (2016) concept of embodied expertise, rehabilitation professionals might integrate outdoor experiences for clients to develop Piran’s (2017) body attunement. Natural environments offer distinctive therapeutic potential, as Caddick et al. (2015) observe that such settings facilitate embodied agency in ways that indoor clinical settings cannot. Outdoor education can challenge its historical privileging of rugged physicality (Warren, 1998) and normative physical capabilities (Laurendeau et al., 2020). Educators might question “what sort of bodies and identities are being produced” (Newbery, 2003, p. 205) in outdoor pedagogical spaces, reimagining disability as a source of knowledge rather than a limitation.

For researchers, this study highlights limitations in traditional body image measures developed for normative bodies (Bailey et al., 2016). New methodological approaches could capture the temporal dimensions of physical capability, recognizing the fluctuating nature of bodily experience and employing participatory approaches that engage women as co-researchers. By centering the embodied knowledge of women experiencing physical challenges, practitioners across fields can create environments where diverse bodies are recognized not as problems but as sources of wisdom and possibility.

## 6. Conclusions: Bodies That Transform

This study extends our understanding of women’s body image by examining how illness, injury, and disability interact with outdoor physical activity to shape body perceptions. By adapting Breault-Hood’s (2023) Holistic Model specifically for diverse physical capabilities, the research provides a framework for understanding body image when navigating physical challenges. Findings reveal the transformative potential of outdoor activities for women experiencing changing physical capabilities. Through engagement with nature, women develop relationships with their bodies characterized by acceptance, gratitude, and redefined functionality, offering guidance for practitioners and directions for future research.

Breault-Hood’s (2023) model proved adaptable and robust, with its eight principles providing sufficient flexibility to accommodate diverse embodied experiences while maintaining theoretical coherence. Participants’ stories offer powerful counter-narratives to cultural norms equating women’s worth with appearance and narrowly defined capabilities. As one participant stated: “My body has experienced a life-threatening illness, and as a result, it is quite different than it used to be. And yet, I can do all that I do, and my body enables me to do it. How could I not love it?”. Theoretically, this adaptation extends feminist disability scholarship by illustrating how outdoor contexts create what Garland-Thomson (2011) describes as productive misfitting—spaces where diverse bodies can develop alternative narratives. For practitioners, these findings suggest approaches for supporting women with diverse physical capabilities by creating accommodating environments, emphasizing personalized functionality, and fostering communities that validate women’s embodied expertise—a shift benefiting all who seek meaningful relationships with their bodies in natural environments.

## Data Availability

The datasets presented in this article are available in the PhD thesis by Joelle Breault-Hood. https://researchers.westernsydney.edu.au/en/studentTheses/living-in-our-bodies (accessed on 21 July 2025).
